# The antifibrotic adipose‐derived stromal cell: Grafted fat enriched with CD74+ adipose‐derived stromal cells reduces chronic radiation‐induced skin fibrosis

**DOI:** 10.1002/sctm.19-0317

**Published:** 2020-06-20

**Authors:** Mimi R. Borrelli, Ronak A. Patel, Sandeep Adem, Nestor M. Diaz Deleon, Abra H. Shen, Jan Sokol, Sara Yen, Erin Y. Chang, Rahim Nazerali, Dung Nguyen, Arash Momeni, Kevin C. Wang, Michael T. Longaker, Derrick C. Wan

**Affiliations:** ^1^ Hagey Laboratory for Pediatric Regenerative Medicine, Department of Surgery, Division of Plastic Surgery Stanford University School of Medicine Stanford California USA; ^2^ Program in Epithelial Biology, Department of Dermatology Stanford University School of Medicine Stanford California USA; ^3^ Stanford Institute for Stem Cell Biology and Regenerative Medicine Stanford University School of Medicine Stanford California USA

**Keywords:** adipose, adipose stem cells, FACS, stem cells, transplantation

## Abstract

Fat grafting can reduce radiation‐induced fibrosis. Improved outcomes are found when fat grafts are enriched with adipose‐derived stromal cells (ASCs), implicating ASCs as key drivers of soft tissue regeneration. We have identified a subpopulation of ASCs positive for CD74 with enhanced antifibrotic effects. Compared to CD74− and unsorted (US) ASCs, CD74+ ASCs have increased expression of hepatocyte growth factor, fibroblast growth factor 2, and transforming growth factor β3 (TGF‐β3) and decreased levels of TGF‐β1. Dermal fibroblasts incubated with conditioned media from CD74+ ASCs produced less collagen upon stimulation, compared to fibroblasts incubated with media from CD74− or US ASCs. Upon transplantation, fat grafts enriched with CD74+ ASCs reduced the stiffness, dermal thickness, and collagen content of overlying skin, and decreased the relative proportions of more fibrotic dermal fibroblasts. Improvements in several extracellular matrix components were also appreciated on immunofluorescent staining. Together these findings indicate CD74+ ASCs have antifibrotic qualities and may play an important role in future strategies to address fibrotic remodeling following radiation‐induced fibrosis.

## Graphical abstract

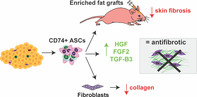

CD74⩲ adipose‐derived stromal cells express multiple growth factors with antifibrotic activity. These cells decrease production of collagen type 1 by TGF‐B1 stimulated fibroblasts, and when used to enrich fat grafts, they promote remodeling of radiation‐induced fibrosis in the overlying skin.CD74⩲ adipose‐derived stromal cells express multiple growth factors with antifibrotic activity. These cells decrease production of collagen type 1 by TGF‐B1 stimulated fibroblasts, and when used to enrich fat grafts, they promote remodeling of radiation‐induced fibrosis in the overlying skin.

**Significance statement**

CD74+ adipose‐derived stromal cells have antifibrotic qualities both in vitro and in vivo and may play an important role in future strategies to address fibrotic remodeling following radiation‐induced fibrosis.

## 1 INTRODUCTION

Radiation‐induced skin fibrosis (RIF) is a complication of radiation therapy (RT) reported in up to 20% of all breast cancer patients.^1^, ^2^, ^3^, ^4^, ^5^, ^6^, ^7^ Characterized by skin pigmentation, reduced elasticity, microvascular obliteration, and dermal thickening,^8^, ^9^ RIF can be painful and disfiguring, and can significantly impair tissue function.^10^ Autologous fat grafting is a surgical technique able to improve the quality of skin damaged by RT.^11^, ^12^, ^13^, ^14^, ^15^ Fat grafting restores a more “normal” skin architecture by decreasing dermal collagen content and overall dermal thickness, creating greater alignment of collagen fiber networks,^9^, ^16^, ^17^ and increasing skin perfusion.^9^, ^17^ The beneficial effects of grafted fat on tissue texture, color, and elasticity cannot be explained through tissue expansion alone,^18^, ^19^ and the adipose‐derived stromal cells (ASCs) within the stromal vascular fraction (SVF) of lipoaspirate are thought to orchestrate tissue regeneration, primarily via the secretion of growth factors with potent adipogenic, angiogenic, and antifibrotic effects.^13^, ^20^, ^21^, ^22^, ^23^, ^24^, ^25^

Recent work has highlighted the existence of multiple distinct subpopulations of stem and progenitor cells contained within the SVF. For example, ASCs with low expression of the surface marker endoglin (CD105)^26^ or those expressing the surface receptor Thy‐1 (CD90)^27^ possess enhanced osteogenic capacities. Similarly, bone morphogenetic protein receptor‐1A marks ASCs with enhanced capacity for adipogenesis,^28^ while endosialin (CD248) characterizes ASCs with angiogenic potential.^29^ Although ASCs have been described to have antifibrotic effects within transplanted tissue, a specific ASC subpopulation characterized by an ability to reduce soft tissue fibrosis has yet to be described. Recent work has highlighted CD74 as a surface marker present on cells with antifibrotic qualities in a number of tissue types. CD74^−/−^ mice show increased liver fibrosis when treated with carbon tetrachloride (CCl_4_) in vivo,^30^ and also develop spontaneous lung injury by 6 months of age.^31^ Given these findings, we hypothesized the existence of a subpopulation of CD74+ ASCs with enhanced antifibrotic actions able to restore irradiated soft tissue defects and modify both critical cell subpopulations and molecular signals involved in radiation fibrosis.

## 2 MATERIALS AND METHODS

### 2.1 Human SVF isolation

Human lipoaspirate samples were obtained from healthy female patients (n = 5) with informed consent under a protocol approved by Stanford Institutional Review Board (IRB #2188). Fat was harvested from the abdomen, flank, and/or thigh under local or general anesthesia using the Coleman technique. The SVF of adipose tissue was isolated as previously described.^17^, ^32^, ^33^ In brief, the lipoaspirate was first washed with 1X sterile phosphate‐buffered saline (PBS, #10010023, Thermo Fisher Scientific, Waltham, Massachusetts), and allowed to sit for 30 minutes at 4°C for separation into layers of blood/debris, fat, and lipid. The layer of fat was retrieved by aspiration and digested using collagenase (Collagenase from Clostridium histolyticum, #C6685, Sigma‐Aldrich, St. Louis, Missouri) dissolved in digest buffer (0.75 mg/mL) of 5% fetal bovine serum (FBS, Gibco, #10082147, ThermoFisher), 100 U/mL DNase I (Worthington, Lakewood, New Jersey), 0.1% Poloxamer 188 (#P5556‐100ML, Sigma‐Aldrich), 20 mM HEPES (#15630080, Sigma‐Aldrich), and 1 mM CaCl_2_, in Medium 199 (#SH30223.02, HyClone, GE Healthcare, Chicago, Illinois) for 30 minutes at 37°C in a shaking water bath (150 rpm). The enzyme was then quenched using fluorescence‐activated cell sorting (FACS) buffer (PBS with 2% FBS, 1 mM EDTA [#15575020, Invitrogen, ThermoFisher], and 1% penicillin‐streptomycin solution [Pen‐Strep, PS, #15140122, ThermoFisher]), filtered through a 100 μm nylon cell strainer, and centrifuged (450*g*, 5 minutes, 4°C). The supernatant was aspirated and the SVF pellet was resuspended in 500 μL of FACS buffer and carefully placed onto Histopaque (Histopaque‐1077 sterile‐filtered, density: 1.077 g/mL, #10771, Sigma Aldrich) by pipette, and then centrifuged at 1500 rpm (27°C, 30 minutes, no deceleration), to remove the red blood cells and cellular debris. The SVF cell layer (buffy coat) was then retrieved, washed in FACS buffer, and centrifuged (450*g*, 5 minutes, 4°C) to leave the purified SVF cell pellet.

### 2.2 Flow cytometry and cell sorting

Human ASCs were isolated from the SVF according to surface marker expression profiles by FACS. The SVF pellet was resuspended in 100 μL for staining with the following anti‐human fluorescent antibodies: CD45‐Pacific Blue (PB) (#304029, Biolegend, San Diego, California) to label hematopoietic cells; CD235a(Glycophorin A)‐eFluor450 (#48‐9987‐42, eBioscience, San Diego, California) to label erythrocytes and erythroid progenitors; CD31(PECAM‐1)‐eFluor450 (#48‐0319‐42, eBioscience) to label endothelial cells; CD34‐Fluorescein Isothiocyanate(FitC) (#555821, BD Biosceinces, San Jose, California) to label ASCs; and CD74‐Allophycocyanin(APC) (#326811, BioLegend) to label the antifibrotic subset of ASCs. All primary antibodies were diluted 1:100. After 30 minutes incubation with primary antibodies shielded from light, cell suspension solutions were diluted with FACS buffer, centrifuged (450*g*, 5 minutes, 4°C), resuspended in 500 μL of FACS buffer, and filtered through a 70 μm nylon mesh. 4',6‐diamidino‐2‐phenylindole (DAPI) (#62248, ThermoFisher) was added as a live‐dead stain (1:10 000). FACS (BD II Aria) was then used to isolate ASCs (CD45−, CD235a−, CD31−, CD34+ live single cells) that were CD74+ (CD45−, CD235a−, CD31−, CD34+, CD74+), CD74− (CD45−, CD235a−, CD31−, CD34+, CD74−), and “unsorted” (US, CD45−, CD235a−, CD31−, CD34+ only).

### 2.3 Gene expression

CD74+, CD74−, and US ASCs were FACS sorted directly into TRIzol lysing solution (#15596026, ThermoFisher) and immediately frozen in dry ice and kept at −80°C until processing. RNA was harvested using RNeasy Mini Kit (#74104, Qiagen, Hilden, Germany). Reverse transcription was performed using TaqMan Reverse Transcription Reagents (#4304134, Invitrogen, ThermoFisher) and an ABI Prism 7900HT Sequence Detection System (#4317596, ThermoFisher) was used to perform quantitative real‐time polymerase chain reaction to evaluate expression levels for genes known to be associated with antifibrotic activity—hepatocyte growth factor (HGF), fibroblast growth factor 2 (FGF2), and transforming growth factor β3 (TGF‐β3)—and the pro‐fibrotic growth factor TGF‐β1. All experiments were run in triplicate and data were standardized to glyceraldehyde 3‐phosphate dehydrogenase expression for statistical analysis. Significant differences in gene expression levels between the CD74+, CD74−, and US ASCs were determined using the relative threshold cycle method.^34^ To obtain ΔCT values, averaged CT values of the reference transcripts were subtracted from CT values of the candidate transcripts. ΔCT values of each gene in the analysis were compared to determine statistically significant differences.

### 2.4 Stimulated collagen production

FACS‐sorted CD74+, CD74−, and US ASCs were directly plated (10 000 cells/gelatin [0.1%]‐coated 24‐well) and expanded at low oxygen conditions in growth media until 4 million cells under passage 3 were obtained. Culture media was then collected for use as conditioned media on human dermal fibroblasts. Human skin was digested to obtain primary cultures of human dermal fibroblasts. Human skin samples were obtained from healthy patients (n = 3) with no underlying comorbidities. Informed consent was obtained under a protocol approved by Stanford Institutional Review Board (IRB #45219). The skin was first washed in PBS and cut into 2 mm thick strips using scissors and submerged in Dispase (25 units/mL [#CB40235, Corning, Fisher scientific]) in Dulbecco's Modified Eagle Medium (DMEM) GlutMax (#10566‐016, Thermo Fisher Scientific) for 18 hours at 4°C for enzymatic separation of the epidermis from the dermis. The epidermis was then removed using sterile forceps and discarded. The remaining dermal tissue was mechanically digested using sterile curved scissors until uniform consistency was reached, and then enzymatically digested using Collagenase (type IV, 1500 U/mL, #17104019, Fisher Scientific) in Hanks Buffered Saline (HBSS, #14025076, Thermo Fisher Scientific) under constant agitation at 37°C for 30 minutes. FACS buffer was added to neutralize the enzyme (1:1), and the digest was filtered through a 100 μm nylon filter. Cell suspensions were pelleted (200 g, 5 minutes, 4°C), resuspended in 500 μL of FACS buffer, carefully pipetted onto 1 mL of Histopaque, and centrifuged. The “buffy coat” of dermal cells was retrieved by pipette, washed, repelleted, and resuspended in 500 μL of fibroblast media (10% FBS, 1% antibiotic‐antimycotic [Gibco, #15240062], 1% GlutMax in DMEM) and expanded in gelatin (0.1%)‐coated wells at low oxygen conditions (2% O_2_ and 7.5% CO_2_) until confluence in 60 mm wells. All cells were kept below passage 3. The media was then aspirated, washed, and dermal fibroblasts were then incubated with media from the cultured CD74+, CD74−, and US ASCs. An amount of 10 ng/60 mm well of transforming growth factor β1 (TGF‐β1, R&D Systems) was added and protein production was assessed following 24 hours.

### 2.5 Protein expression

FACS isolated CD74+, CD74−, and US ASCs were analyzed for production of TGF‐β3 and TGF‐β1 and dermal fibroblasts were analyzed for production of Procollagen type 1, Collagen type 1, and Collagen type 3 by Western blots. Cells were lysed with RIPA buffer and centrifuged at 10 000*g* for 30 minutes. Proteins were loaded and run on a BioRad 4‐20% mini‐PROTEAN TGX precast gel (BioRad Laboratories, Hercules, California). Proteins were then transferred to an Immobilon‐P membrane and probed overnight at 4°C with primary antibodies in 5% milk (polyclonal rabbit anti‐TGF‐β3 [ab15337, Abcam, Cambridge, United Kingdom; 1:1000], polyclonal rabbit anti‐TGF‐β1 [ab92486, Abcam; 1:1000], polyclonal rabbit anti‐pro‐collagen type 1 [abt257, Sigma‐Aldrich; 1:500], monoclonal mouse anti‐collagen 1 [ab6308, Abcam; 1:1000], monoclonal mouse anti‐collagen 3 [ab6310, Abcam; 1:1000], and monoclonal mouse anti‐beta Tubulin [E7‐S, DSHB, Iowa City, Iowa; 1:2000]). Incubation with an appropriate horseradish peroxidase (HRP)‐linked secondary antibody and enhanced chemiluminescence were used for protein detection (#34075 SuperSignal West Dura Extended Duration Substrate, ThermoFisher).

### 2.6 Mice scalp irradiation and fat grafting

**FIGURE 1:**
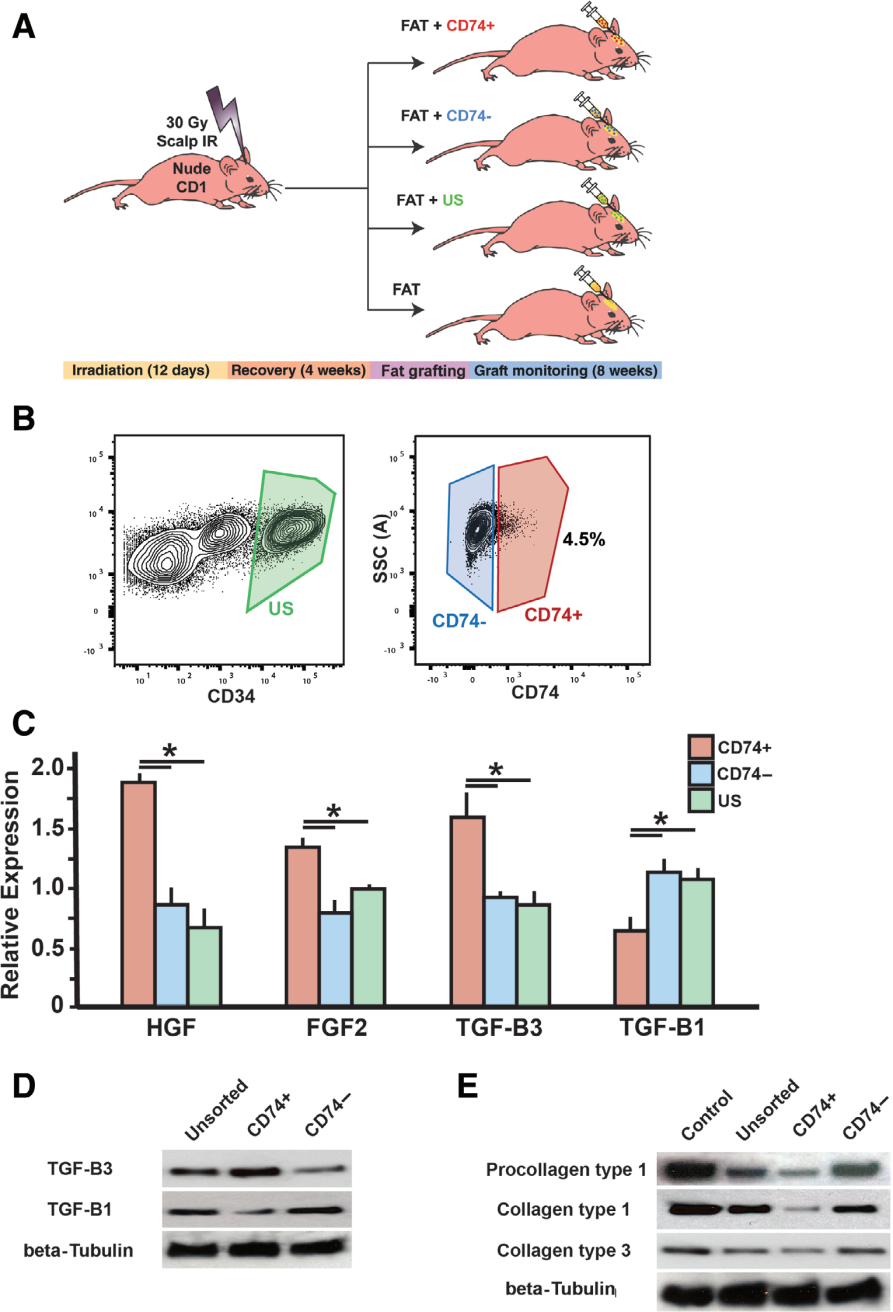
Isolation of adipose‐derived stromal cell subpopulations and analysis of effects. A, Schematic of overall experimental design used to explore antifibrotic effects of CD74+ ASCs within irradiated soft tissue beds. Mice were grafted with fat (n = 5/group): (1) enriched with CD74+ ASCs; (2) enriched with CD74− ASCs; (3) enriched with US ASCs; or (4) not enriched with ASCs. B, Isolation of CD74+ ASCs. Flow cytometry plots showing gating strategy used to isolate CD74+ ASCs. ASCs were defined as CD34+ live single cells, negative of lineage markers CD45, CD235a, and CD31. CD74+ ASCs comprised 4.5% of all ASCs within SVF. C, Quantitative real‐time reverse transcription polymerase chain reaction‐PCR showing differentiation expression of antifibrotic growth factors—HGF, FGF2, and TGF‐β3—and pro‐fibrotic growth factor TGF‐β1. CD74+ ASCs had significantly increased expression of HGF, FGF2, and TGF‐β3 and decreased TGF‐β1 compared to both CD74− ASCs (*P < .05) and US ASCs (*P < .05). D, Representative Western blots of TGF‐β3 (top) and TGF‐β1 (middle) from US, CD74+, and CD74− ASCs with beta‐Tubulin loading control (bottom). E, Representative Western blots of Procollagen type 1 (top), Collagen type 1 (second from top), and Collagen type 3 (third from top) synthesized by stimulated human dermal fibroblasts (control) and fibroblasts incubated in conditioned media from US, CD74+, and CD74−, ASCs with beta‐Tubulin loading control (bottom). ASC, adipose‐derived stromal cell; FGF2, fibroblast growth factor 2; HGF, hepatocyte growth factor; SVF, stromal vascular fraction; TGF‐β1, transforming growth factor β1; TGF‐β3, transforming growth factor β3; US, unsorted

Mice received irradiation to the scalp, using methodology previously described.^9^, ^17^ A total of 30 Gy was delivered, fractionated into six 5 Gy doses on alternate days over a total of 12 days. Lead shielding was used to ensure only the scalp was irradiated. A 4‐week recovery period followed irradiation to allow for the chronic fibrotic effects of radiation to develop (Figure 1A). ASC‐enriched grafts were prepared by mixing 10 000 FACS‐sorted CD74+, CD74−, or US ASCs with 200 μL of fresh human lipoaspirate, based on previous titration studies evaluating effects of supplemental stromal cells.^35^ Control mice received 200 μL of fresh human lipoaspirate not enriched with ASCs (n = 5 mice/group for a total n = 20). Fat was grafted subcutaneously, directly beneath the irradiated scalp skin. A subcutaneous tunnel was first created, and the grafts were delivered by using a 1 cc syringe and a 16‐gauge needle in a retrograde fashion. Fat was injected within 2 hours of original harvest. Injection sites were closed with 6‐0 Vicryl suture (Ethicon, Inc., Somerville, New Jersey). All animal studies were performed in accordance with Stanford University animal guidelines, under the Stanford Institutional Review Board approval (APLAC #31212; Figure S1).

### 2.7 Microcomputed tomography and fat volume rendering

Microcomputed tomography (microCT) was performed immediately following injection and every other week for 8 weeks total. Mice were anesthetized with isoflurane (3% induction, 2% maintenance, inhalation) and imaging was performed with animals in the prone position using the Bruker SkyScan 1276 microCT (Billerica, Massachusetts). The imaging protocol involved the Bruker SkyScan 1276 software (Billerica, Massachusetts). The microCT duration was 73 seconds with a voxel resolution of 41.1 μm and a relative mouse irradiation dose of 64 mGy. Data were subsequently reconstructed into cross sections using the Bruker NRecon software (Billerica, Massachusetts) in blinded fashion to each treatment group. Cross‐sectional images were analyzed for volume using the Bruker CTAn software (Billerica, Massachusetts) by limiting to sections with the fat graft. On the cross‐sectional images, fat could be distinguished from other tissues by gating −500 to 500 Hounsfield units. A region of interest was drawn surrounding the fat graft every 10 slices for the length of the graft. Using three‐dimensional (3D) cubic spline interpolation, a resulting 3D region of interest was generated, and the volume measured.^36^ 3D surfaces were rendered using the Bruker CTVol software (Billerica, Massachusetts) by gating −500 to 500 for fat and 3500 to 10 000 for bone. All analyses were performed by a single person (R. A. P.) blinded to the experimental group of the mice, in order to eliminate interuser variability.^37^

### 2.8 Skin mechanical strength testing of irradiated skin

Eight weeks postgrafting, the mice were sacrificed and the full‐thickness scalp skin overlying the grafted fat was harvested for mechanical strength testing (MST), histological assessment of skin structure, and FACS‐sorting of fibroblast subpopulations. MST was performed using methodology previously described.^7^ In brief, the skin tissue was attached to custom grips of a microtester (model 5848, Instron, Norwood, Massachusetts) equipped with a 100 N load cell using double‐sided tape to provide a gauge length of 1 cm. The tissue specimen was stretched until a break in the skin was detected, observed as a decrease in stress despite increasing strain. Change in length divided by gauge length was used to calculate true strain. True stress was determined by dividing force by the original tissue cross‐sectional area. Ultimate tensile strength corresponds to the greatest true stress achieved prior to breakage.

### 2.9 Histological staining of irradiated skin and fat explants

The skin and explanted fat explants were immediately fixed in 4% paraformaldehyde for 16 hours at 4°C. Samples were then washed with PBS, dehydrated in gradients of alcohols, and embedded in paraffin blocks. Blocks were sectioned into 8‐μm slices and fat was stained with H&E (#H‐3502, Vector Laboratories, Burlingame, California) for assessment of integrity, and skin was stained with H&E to assess dermal thickness and with Masson's Trichrome (#HT15‐1KT, Sigma Aldrich) and Picrosirius Red to determine collagen content. Slides were imaged using a Leica DM5000 B Light microscope (Leica Microsystems, Buffalo Grove, Illinois) using a ×10 objective. Dermal thickness was defined as the distance from the epidermis to the hypodermis, and measurements were made on 10 stained samples from each specimen using image J software (https://imagej.nih.gov/ij/, NIH, Bethesda, Maryland). Collagen content was determined using ImageJ based upon pixel‐positive area per high power field using the same intensity threshold for all images. Five measurements were made per sample, and the mean of the total 10 measurements per sample was recorded as the value for that sample. Images of H&E‐stained fat explants were imaged using a ×20 objective, and 10 random sections were chosen for each mouse for each group for histological analysis and scoring. Four blinded, independent investigators (M. R. B., R. A. P., A. H. S., N. M. D. D.) evaluated fat graft quality in terms of: (a) integrity (presence of intact, nucleated fat cells); (b) cyst/vacuoles; (c) inflammation; and (d) fibrosis, following the methods of a previously published protocol.^38^

### 2.10 Immunostaining

The skin was also prepared for immunostaining by fixing in 4% paraformaldehyde for 16 hours at 4°C. Samples were then washed with PBS and soaked in 30% sucrose in PBS for 3 to 5 days in preparation for embedding. Tissue blocks were prepared by embedding in Tissue‐Tek O.C.T. (Tissue‐Tek* O.C.T Compound, 25608‐930) frozen in a dry ice/ethanol bath. Frozen blocks were sectioned at a thickness of 8 μm and then transferred to Superfrost Plus microscope slides (Fisherbrand). Slides were treated with a blocking reagent (HK083‐5 K, Power Block, BioGenex) and then incubated for 1 hour at room temperature with primary anti‐mouse antibodies against elastin (ab21610, Abcam), fibrillin (ab53076, Abcam), and versican (MA5‐27638, ThermoFisher). Specimens were then washed in PBS, incubated with Alexa‐dye conjugated secondary antibodies for 1 hour at 37°C, and mounted in DAPI Fluromount‐G (#00‐4959‐52, Invitrogen). Fluorescent images were taken using the LSM 880 inverted confocal microscope (Airyscan, GaAsP detector, 880, Beckman) and quantified by determining percent positive pixel counts per high power field.

### 2.11 FACS analysis of fibroblast subpopulations within the irradiated skin

To explore the effect of fat grafts on the fibroblast subpopulations within the overlying skin, mouse skin was digested and prepared for FACS analysis as described above. Cell suspensions were stained with the following anti‐mouse fluorescent antibodies: CD45‐PB (#103125, BioLegend) to label primary hematopoietic cells; Ter‐119‐PB (#116231, BioLegend) to label erythrocytes and erythroid progenitors; Tie‐2(CD202b)‐Biotin (#124005, BioLegend) to label pericytes; CD324(E‐Cadherin)‐Biotin (#324120, BioLegend) and CD326(EpCAM, epithelial cell adhesion molecule)‐Biotin (#118203, BioLegend) to label epithelial cells; CD31‐PB (#102421, BioLegend) to label endothelial cells; Dlk‐1‐APC (#FAB8634A‐025, R&D Systems, Minneapolis, Minnesota); Sca‐1(Ly‐6A/E)‐Brilliant Violet(BV)605 (#108133, BioLegend), CD26‐PerCP/Cyanine(Cy)5.5 (#100623, BioLegend), and platelet derived growth factor receptor alpha (PDGFRα, CD140a)‐PE‐Cy7 (#135911, BioLegend) to label fibroblasts. All primary antibodies were diluted (1:100) in FACS buffer and incubated for 30 minutes at 4°C. Cell suspensions were then diluted in FACS buffer, centrifuged (450*g*, 5 minutes, 4°C), and resuspended in 100 μL of FACS buffer for staining with eFluor 450‐conjugated streptavidin (#48‐4317‐82, Thermofisher) on ice for a further 20 minutes. Samples were then washed in FACS buffer, centrifuged (450*g*, 5 minutes, 4°C), resuspended in 500 μL for FACS, filtered through a 70 μm nylon mesh, and stained with DAPI as a live‐dead stain to determine by FACS the relative percentages of the fibroblast subpopulations (all PDGFRα+Lin‐Live single cells): papillary (CD26+Sca−), reticular (Dlk+Sca−), lipofibroblast (Dlk+Sca+), and zigzag (CD26−) (Figure S2).^39^

### 2.12 Statistical analysis

Data are presented as means ± SD, depicted by error bars. GraphPad Prism (GraphPad Software, La Jolla, California) was used to perform all statistical analyses. Differences between means were compared by analysis of variance (ANOVA) and Bonferroni correction for post hoc group comparisons. A *P* value of <.05 was considered statistically significant.

## 3 RESULTS

### 3.1 CD74 marks an antifibrotic subset of ASCs

Flow cytometry of fresh human lipoaspirate (n = 5) confirmed existence of a subpopulation of CD74+ ASCs that comprised almost 5% of the SVF (Figure 1B). Compared to CD74− ASCs and US ASCs, the CD74+ ASCs had significantly increased expression of HGF, FGF2, and TGF‐β3 (all *P < .05), growth factors with potent antifibrotic actions (Figure 1C).^40^, ^41^, ^42^ Conversely, TGF‐β1 expression was found to be lower in CD74+ ASCs compared to CD74− and US cells (Figure 1C). These findings were confirmed by Western blot which revealed greater TGF‐β3 protein and decreased TGF‐β1 protein levels with CD74+ ASCs (Figure 1D). Primary cultures of human dermal fibroblasts incubated in conditioned media from CD74+ ASC exhibited decreased production of procollagen type 1 as well as collagen type 1 upon stimulation with TGF‐β1 (Figure 1E). Though less marked, Collagen type 3 production was also found to be decreased with CD74+ ASC conditioned media (Figure 1D). These data are consistent with potential antifibrotic activity by CD74+ ASCs which may be mediated by paracrine signaling.

### 3.2 CD74+ ASCs improve retention and the histological quality of grafted fat

**FIGURE 2:**
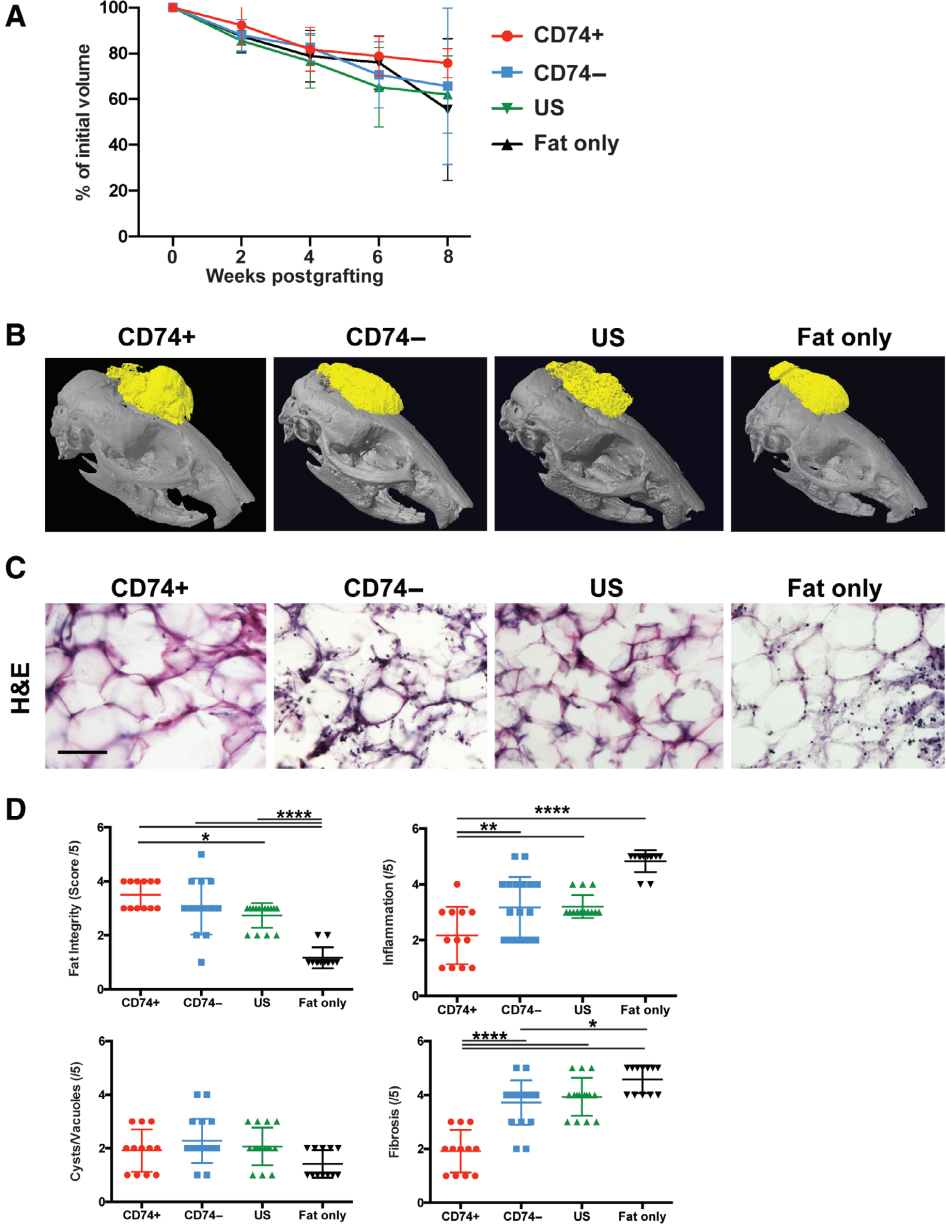
CD74+ ASC‐enrichment improves fat graft quality. A, Average volume retention, expressed as a percentage with respect to baseline volume, measured over 8 weeks. B, Representative reconstructed microcomputed tomography images of enriched fat and fat only at 8‐weeks postgrafting. There was a trend toward greater fat retention in mice receiving grafts of fat enriched with CD74+ ASCs, compared to fat grafts enriched with CD74− or US ASCs and fat alone. C, Representative H&E‐staining of explanted fat grafts 8 weeks after implantation at ×10 magnification. D, Scoring of graft architectural characteristics based on H&E‐stained sections. Explanted fat grafts enriched with CD74+ ASCs had greater integrity (****P < .001), less inflammation (****P < .0001), and were less fibrotic (****P < .001) compared to the fat grafts enriched with CD74− or US ASCs and fat alone. Scale bar = 100 μm. *P < .05; **P < .01; ****P < .0001. ASC, adipose‐derived stromal cell; US, unsorted

To explore the antifibrotic potential of CD74+ in vivo within irradiated tissue, the irradiated scalp of female 60‐day‐old (Adult) CD1‐Nude mice (Crl:CD1‐Foxn1^nu^, Charles River Laboratories International, Inc., Hollister, California) with chronic fibrosis was grafted with fat: (a) enriched with CD74+ ASCs; (b) enriched with CD74− ASCs; (c) enriched with US ASCs; or (d) not enriched with ASCs (fat alone) (n = 5/group) (Figure 1A). Radiographic monitoring of the fat grafts over 8 weeks showed a tendency toward greater retention of grafts enriched with CD74+ ASCs, compared with fat grafts enriched with CD74− and US ASCs or fat alone (Figure 2A,B). The fat grafts were then explanted for histological assessment, which showed grafts enriched with CD74+ ASCs had greater integrity (****P < .001), reduced inflammation (****P < .0001), and were less fibrotic (****P < .001) than fat grafts enriched with CD74− and US ASCs or fat alone (Figure 2C,D).

### 3.3 Fat grafts enriched with CD74+ ASCs reduced fibrosis in overlying skin

**FIGURE 3:**
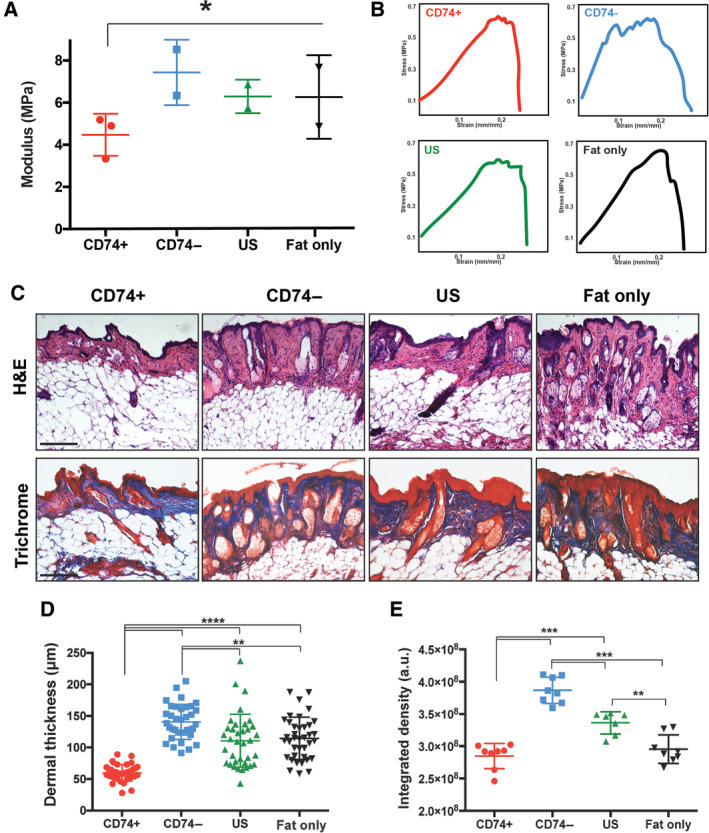
CD74+ ASC‐enriched fat grafts improve radiation‐injured skin. A, Tensile strength of irradiated skin overlying enriched and unenriched fat grafts with calculated Young's modulus (*P < .05), and, B, Representative stress‐strain curves demonstrating measurement of Young's Modulus showing that skin overlying fat enriched with CD74+ ASCs was less stiff than skin in mice of all other groups. C, Histological assessment of skin stained with H&E (top) and Masson's Trichrome (bottom). Representative images are shown at ×20 magnification. D, Dermal thickness of skin overlying fat grafts enriched with CD74+ ASCs was significantly thinner (****P < .0001) and (E) had significantly less collagen (***P < .001) than the skin overlying fat grafts enriched with CD74− and US ASCs and fat alone at week 8. Scale bar = 100 μm. **P < .01; ***P < .001; ****P < .0001. ASC, adipose‐derived stromal cell; US, unsorted

To explore whether fat grafts enriched with CD74+ ASCs had an antifibrotic influence on surrounding irradiated skin and soft tissue, the skin overlying the fat grafts was harvested for biomechanical testing and histological assessment 8‐weeks postgrafting. Calculation of Young's modulus indicated that the skin overlying fat grafts enriched with CD74+ ASCs was less stiff (*P < .05) (Figure 3A,B), had reduced dermal thickness (****P < .0001) (Figure 3C,D), and had significantly less collagen (***P < .001) (Figure 3C,E) than the skin of mice receiving fat grafts enriched with CD74− and US ASCs or fat alone.

### 3.4 Fat grafts enriched with CD74+ ASCs reduced fibrosis in overlying skin

**FIGURE 4:**
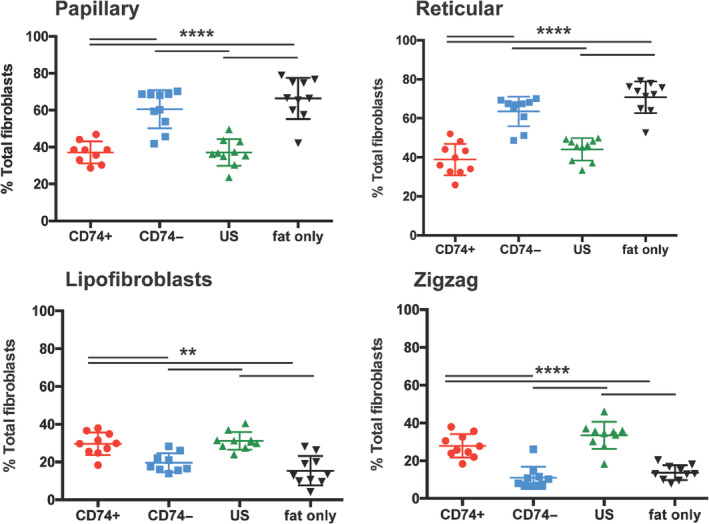
Dermal fibroblast subpopulation composition in irradiated skin overlying fat grafts. There were significantly fewer papillary (top left) (****P < .0001) and reticular (top right) (****P < .0001) fibroblasts, and significantly greater lipofibroblasts (bottom left) (**P < .01) and zigzag (bottom right) (****P < .0001) fibroblasts in skin overlying CD74+ ASC‐enriched fat compared to skin overlying fat enriched with CD74− and US ASCs or fat alone. ASC, adipose‐derived stromal cell; US, unsorted

Recent work investigating fibroblast heterogeneity has highlighted the existence of distinct fibroblast subpopulations with diverse roles. Papillary and reticular fibroblasts are both hypothesized to be pro‐fibrotic. Papillary fibroblasts are positive for CD26, a surface marker associated with fibroblasts with pro‐fibrotic phenotype in the mouse dorsal dermis,^7^ and reticular fibroblasts reside deep within the dermis,^39^ the location of maximal scar production. Lipofibroblasts and zigzag fibroblasts, on the other hand, are hypothesized to be more antifibrotic; lipofibroblasts are adipocyte‐precursors, and fat is recognized for its ability to minimize scars^19^, ^43^, ^44^, ^45^ and soft tissue fibrosis.^44^, ^46^, ^47^, ^48^ Zigzag fibroblasts are located at the base of hair follicles in healthy nonfibrotic skin but are absent in scars. To explore the influence of grafted fat enriched with CD74+ ASCs on the cellular composition of overlying irradiated skin, FACS was used to assess the relative proportions of these four fibroblast subpopulations in the skin overlying grafted fat enriched with CD74+, CD74−, US ASCs, and fat alone. Interestingly, CD74+ ASC‐enriched fat decreased the proportion of the more fibrotic papillary (****P < .0001) and reticular (****P < .0001) fibroblasts, and significantly increased the proportions of the more antifibrotic zigzag (****P < .0001) and lipofibroblasts (**P < .01), compared to skin overlying fat enriched with CD74− fat or fat alone (Figure 4). These results are consistent with an antifibrotic effect of CD74+ ASCs within grafted fat on the surrounding soft tissue at recipient sites.

### 3.5 Fat grafts enriched with CD74+ ASCs promote regeneration of elastic fibers

**FIGURE 5:**
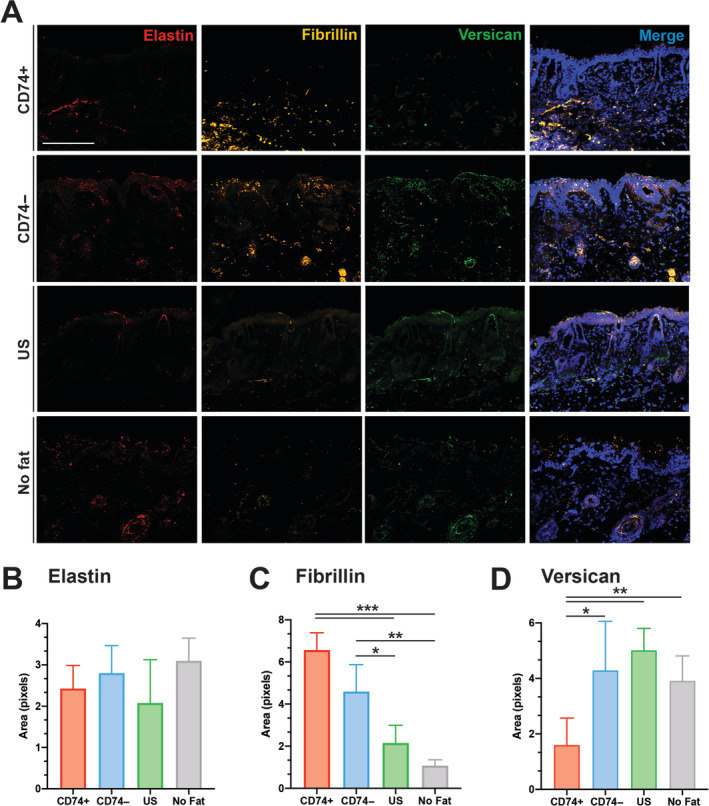
Immunofluorescence staining for elastic fibers. A, Staining for elastin (red, far left column), fibrillin (yellow, second column), and versican (green, third column) highlights how fat grafting alters components of irradiated extracellular matrix. Merged image with 4',6‐diamidino‐2‐phenylindole (DAPI) counterstain (blue) on the far‐right column. B, Pixel positive‐percent quantification of elastin staining, C, fibrillin staining, and, D, versican staining. Note mice grafted with fat enriched with CD74+ ASCs had improvement in staining for fibrillin (***P < .001, **P < .01, *P < .05), with a concomitant decrease of versican (**P < .01, *P < .05). Scale bar = 100 μm. ASC, adipose‐derived stromal cell

Radiation is known to alter several different fibers within the extracellular matrix.^36^ To explore whether fat grafting had a beneficial effect on these components in addition to the collagen content and dermal thickness, we stained for elastin, fibrillin, and versican. While we observed no significant change in elastin fibers between any of the groups, there were notable differences in fibrillin and versican fibers. Radiation is known to impact the integrity of fibrillin fibers which interacts with TGF‐β1.^49^, ^50^ Importantly, mice grafted with fat enriched with CD74+ ASCs had the greatest improvement in fibrillin staining relative to irradiated soft tissue (***P < .001) **(**Figure 5 **)**. Immunofluorescent staining for versican, a pro‐fibrotic modulator, was also decreased with CD74+ ASC‐enriched fat compared to all other groups (*P < .05, **P < .01) (Figure 5).

## 4 DISCUSSION

Significant soft tissue fibrosis following RT can distort skin form, impair skin function, and negatively impact patient quality‐of‐life.^1^, ^2^, ^3^, ^4^, ^5^, ^6^ Despite these negative consequences, RT remains an important anticancer treatment, and is used to cure or palliate over 50% of cancer patients.^51^, ^52^ With increasing numbers of patients surviving cancer and increasing risks of patients experiencing the long‐term effects of RT, it is of paramount importance to prevent or reverse the pathological fibrotic process.^22^, ^53^ Fat grafting can improve the quality, hyperpigmentation, and thickness of irradiated skin.^8^, ^11^, ^12^, ^13^, ^14^, ^15^ ASCs within the grafted fat are thought to drive tissue regeneration, and recent work has identified numerous subpopulations of ASCs with distinct properties.^26^, ^27^, ^28^, ^29^ Here, we describe an additional subpopulation of ASCs, positive for the surface marker CD74, with enhanced antifibrotic qualities.

CD74 is a nonpolymorphic type II transmembrane glycoprotein which functions as a major histocompatibility complex class II chaperone and has a high affinity for the macrophage inhibitory factor (MIF) receptor. CD74 is thought to be anti‐inflammatory via its interaction with MIF; in hematopoietic stem cells MIF binds CD74, and this instigates intracellular signaling culminating in phosphorylation of AMP‐activated protein kinase (AMPK). AMPK, in turn, inhibits platelet derived growth factor induced migration and proliferation of hepatic stellate cells.^54^ This pathway may also mediate the antifibrotic activity of cells known to express the CD74 surface marker in other tissues, such as adipose tissue.

In our study, we observed ASCs positive for the surface marker CD74 to have antifibrotic qualities. Though this may derive, in part, from an anti‐inflammatory function, we also found CD74+ ASCs to express antifibrotic genes and media from cultured CD74+ ASCs reduced collagen production, particularly collagen type 1 which has been shown to be significantly increased relative to collagen type III in fibrosis/scar,^55^ in stimulated human dermal fibroblasts in vitro. Fat grafts enriched with CD74+ ASCs had greater improved histological quality, underwent less resorption, and had an antifibrotic influence on surrounding irradiated soft tissue. Specifically, CD74+ ASC‐enriched fat grafts decreased stiffness, thickness, and collagen content of overlying skin, and decreased the proportions of fibrotic fibroblast subpopulations. Furthermore, this was also associated with enhanced staining for fibrillin which may play a role in modulation of TGF‐β1 activity. Fibrillin may interact with latent TGF‐β1^50^ and murine knockouts of fibrillin‐1 have been found to have greater interstitial fibrosis secondary to increased TGF‐β1 activation.^56^, ^57^ Finally, versican, a chondroitin sulfate proteoglycan known to promote fibrogenic cellular functions,^58^ was noted to be lower in irradiated soft tissue grafted with CD74+ ASC‐enriched fat compared to CD74− ASC or US ASC‐enriched fat.

The transforming growth factor beta (TGF‐β) superfamily are critical regulators of tissue repair and fibrosis and three isoforms of TGF‐β (TGF‐β1, 2, and 3) are known to possess distinct roles.^59^ Interestingly, all TGF‐β isoforms act through the same receptors, suggesting antagonizing functions.^59^ Differential activation of downstream small mothers against decapentaplegic (SMAD) signaling intermediates may also contribute to their contrasting functions. In particular, recent studies have shown Smad7 to suppress fibrosis in multiple organs, and further exploration of this may be warranted with respect to our observations with CD74+ ASCs.^60^, ^61^ In our study, we found that CD74+ ASCs express greater levels of TGF‐β3, the isoform with the most antifibrotic activity,^62^ and had decreased levels of TGF‐β1 transcripts, which is known to mediate fibrosis.^63^ Supporting our findings, recent reports have shown that ASCs can inhibit fibroblast proliferation by decreasing TGF‐β1 expression, and promote collagen remodeling by increasing TGF‐β3.^64^, ^65^ Furthermore, connective tissue growth factor has been implicated as a cofactor with TGF‐β1 in mediating fibrosis,^66^, ^67^ and it may be of interest to evaluate the impact CD74+ ASCs may play in production of this growth factor in subsequent studies.

Identification and isolation of ASC subpopulations with antifibrotic potential can both expand the current understanding of adipose tissue biology and expedite the application of specific ASC subpopulations for therapeutic benefit. While enrichment of fat grafts with ASCs (cell‐assisted lipotransfer or CAL) can improve retention rates, enhance the quality of grafted fat, and further attenuate radiation‐induced dermal thicknening^9^, ^68^, ^69^, ^70^, ^71^, ^72^ compared to fat alone, we demonstrate here that these effects are more pronounced when grafted fat is enriched with CD74+ ASCs, relative to CD74− or US ASCs. Thus CD74+ ASCs may have a potentially important role in the treatment of radiation‐induced soft tissue fibrosis. While the CD74+ subpopulation comprised a small fraction of the SVF and may require substantial in vitro expansion prior to for grafting in the clinical setting, one option may be to expand or enhance the activity of the CD74+ within lipoaspirate in vivo using targeted molecules. And aside from this consideration, our findings of a potential role CD74+ ASCs may play in improving fibrosis also help to begin explaining the regenerative effects of fat grafting already observed clinically.^8^, ^11^

## 5 CONCLUSION

In summary, CD74 marks a subpopulation of ASCs with antifibrotic qualities both in vitro and in vivo. CD74+ ASCs may attenuate production of pro‐fibrotic extracellular matrix components by fibroblasts and promote improvement of detrimental histologic and biomechanical changes to skin following radiation injury. The findings in our mouse study thus raise the potential for use of lipoaspirate enriched with CD74+ ASCs in the clinical setting to reduce the damaging effects of RT.

## CONFLICT OF INTEREST

N.M.D.D. declared research funding from California Institute for Regenerative Medicine. R.N. declared consultant/advisory role with Mentor Worldwide, Musculoskeletal Transplant Foundation, Telabio, Inc. A.M. declared consultant/advisory for Allergan, AxoGen, Sientra, and Stryker and research funding from AxoGen. The other authors declared no potential conflicts of interest.

## AUTHOR CONTRIBUTIONS

M.R.B., R.A.P.: contribution to designing research studies, and conducting experiments and acquiring data, data analysis, manuscript writing and final approval of the manuscript; A.M.: contribution to designing research studies, and conducting experiments and acquiring data; K.C.W., M.T.L.: contribution to designing research studies, manuscript writing and final approval of the manuscript; D.C.W.: contribution to designing research studies, and conducting experiments and acquiring data, manuscript writing and final approval of the manuscript; S.A., N.M.D.D., A.H.S.: contribution to conducting experiments and acquiring data, data analysis, manuscript writing and final approval of the manuscript; J.S., S.Y., E.Y.C.: contribution to conducting experiments and acquiring data, data analysis; R.N., D.N.: contribution to conducting experiments and acquiring data.

## Supporting information


**Fig. S1** APLAC protocol #31212 approval used for mouse irradiation experiments.

**Fig. S2** **Gating strategy used to isolate dermal fibroblast subpopulations by fluorescence‐activated cell sorting.** A negative (lineage‐) and positive (PDGFRa+) gating strategy was used to isolate the four fibroblast subpopulations: papillary (CD26 + Sca‐), reticular (Dlk + Sca‐), Lipofibroblast (Sca+), and zigzag (CD26‐).

## Data Availability

The data that support the findings of this study are available on request from the corresponding author.
