# Supplementary figures and images for: The antifibrotic adipose‐derived stromal cell: Grafted fat enriched with CD74+ adipose‐derived stromal cells reduces chronic radiation‐induced skin fibrosis
Source: Stem Cells Transl Med. 2020 Jun 20;9(11):1401–13. doi: 10.1002/sctm.19-0317 (PMC7581454; doi:10.1002/sctm.19-0317)

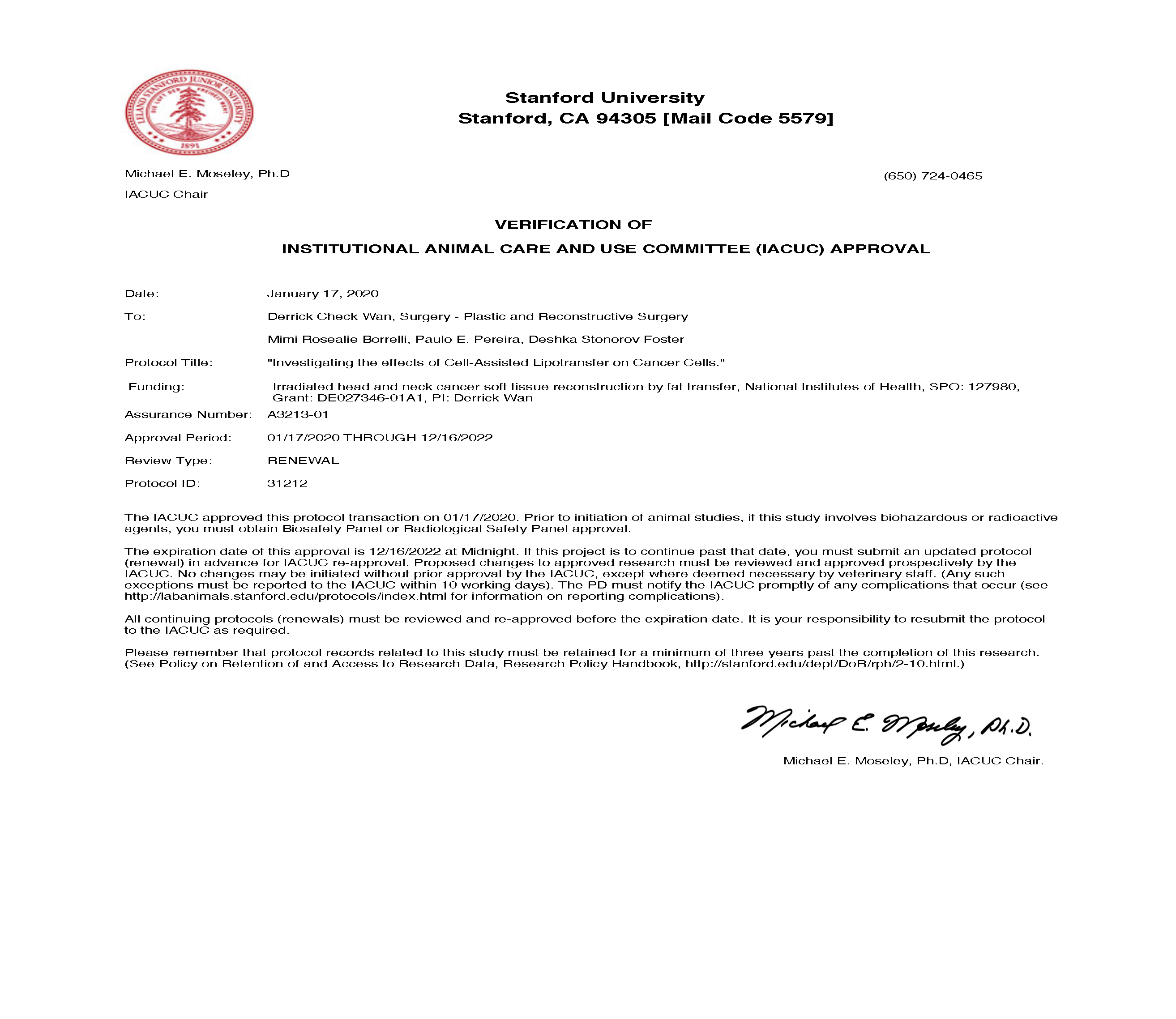

Supplement: Supplementary file 1 — Fig. S1 APLAC protocol #31212 approval used for mouse irradiation experiments. [file SCT3-9-1401-s001.tif]

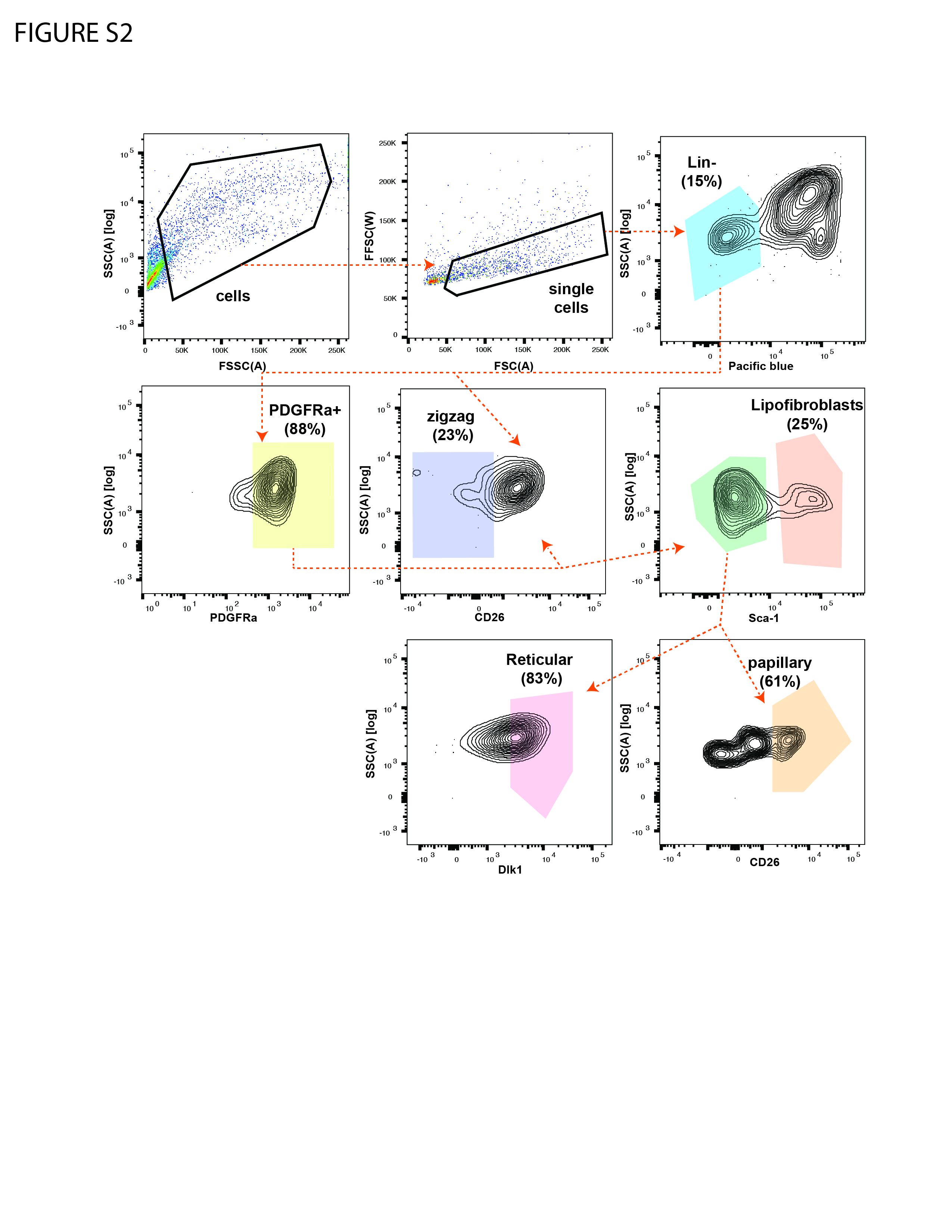

Supplement: Supplementary file 2 — Fig. S2 Gating strategy used to isolate dermal fibroblast subpopulations by fluorescence‐activated cell sorting. A negative (lineage‐) and positive (PDGFRa+) gating strategy was used to isolate the four fibroblast subpopulations: papillary (CD26 + Sca‐), reticular (Dlk + Sca‐), Lipofibroblast (Sca+), and zigzag (CD26‐). [file SCT3-9-1401-s002.tif]
